# Usefulness of the Pain Tracking Technique in Acute Mechanical Low Back Pain

**DOI:** 10.1155/2015/512673

**Published:** 2015-07-09

**Authors:** Tania Bravo Acosta, Jorge E. Martín Cordero, Solangel Hernández Tápanes, Isis Pedroso Morales, José Ignacio Fernández Cuesta, Maritza Leyva Serrano

**Affiliations:** ^1^Clinical Research Center, Playa, Havana, Cuba; ^2^Medical-Surgical Research Center, Playa, Havana, Cuba; ^3^Luis de la Puente Uceda Hospital-Polyclinic, 10 de Octubre, Havana, Cuba; ^4^Public Health Ministry, Plaza, Havana, Cuba

## Abstract

*Objective*. To evaluate the usefulness of the pain tracking technique in acute mechanical low back pain. *Method*. We performed an experimental prospective (longitudinal) explanatory study between January 2011 and September 2012. The sample was randomly divided into two groups. Patients were assessed at the start and end of the treatment using the visual analogue scale and the Waddell test. Treatment consisted in applying the pain tracking technique to the study group and interferential current therapy to the control group. At the end of treatment, cryotherapy was applied for 10 minutes. The Wilcoxon signed-rank test and the Mann Whitney test were used. They were performed with a predetermined significance level of *p* ≤ 0.05. *Results*. Pain was triggered by prolonged static posture and intense physical labor and intensified through trunk movements and when sitting and standing. The greatest relief was reported in lateral decubitus position and in William's position. The majority of the patients had contracture. Pain and disability were modified with the rehabilitation treatment in both groups. *Conclusions*. Both the pain tracking and interferential current techniques combined with cryotherapy are useful treatments for acute mechanical low back pain. The onset of analgesia is faster when using the pain tracking technique.

## 1. Introduction

Low back pain is a common ailment, so much so that it is estimated that 80% of the population will suffer from it at some point in their lives [1–6].

It is thought that 30% of adolescents have experienced at least one episode of low back pain. However, first episodes most frequently present themselves in adults aged between 20 and 40, dropping in middle age, just when X-ray and imaging alterations become more evident. This supports the hypothesis that casts doubt on the relationship between disc lesions and low back pain, with or without pain radiation [7].

Low back pain is the second leading cause for GP consultations, the fifth for hospitalisation, and the third for surgical intervention of the musculoskeletal disorders. It is the third cause of chronic functional disability, after respiratory complaints and trauma. It has been found that low back pain has a high prevalence, independently of the socioeconomic status of a given population [8, 9].

It can be characterised by presenting low back pain in the vertebral or paravertebral region, with radiation to the gluteal region and down to the middle third of the rear of both thighs. It increases through physical activity and certain movements and is relieved through rest and certain antalgic positions. It does not wake the patient during the night. Frequently, there is a trigger, there have been previous episodes, and there are no associated symptoms. This pain generally corresponds to a structural alteration or functional-postural overloading of the elements that make up the lumbar spine, namely, the vertebral body, ligaments, intervertebral discs, and paravertebral muscles, meaning that aetiological diagnosis is only possible in a small percentage of cases. It is also the reason why the majority of instances of low back pain of this kind fall into the category of nonspecific low back pain [1].

Prevention is one of the fundamental aspects in the treatment of low back pain, with a view to limiting its serious socioeconomic repercussions. It is based on three central pillars: postural hygiene, exercises, and lifting weights in inadequate postural positions [10, 11]. Back schools run educational and skills acquisition programmes whose main objective is to inform patients and foster individual responsibility for low back pain. Benefits are reported for cases of low back pain lasting over 2 weeks, though not for pain lasting over 12, with evidence level B [12–14].

As regards traction, a Spanish work group concluded that the effectiveness of traction is unknown (level D evidence) and in fact proved no more effective than the placebo (level C evidence) [12, 13].

There are very few meta-analyses or controlled clinical trials into the usefulness of physical agents in low back pain [15–18]. Among those that can be prescribed is heat therapy [19–21]. French et al., in their systematic review of low back pain, conclude that moderate evidence exists in which damp hot compresses provide a small amount of relief on the short term in people with acute and subacute low back pain [19]. Other studies specify level 1b evidence and recommendation A, given their positive effects on pain, rigidity, and disability [20].

Current practice sees ice recommended during the first 72 hours, though there is insufficient evidence of its effects in patients with acute or subacute pain [19, 20].

The use of laser has been studied, concluding that no study has been carried out to evaluate its use in the treatment of low back pain [22].

The above measures provide satisfactory relief to a large number of patients. However, there remains a group that reports no pain relief [23].

Electrotherapy is considered among the noninvasive pain treatment techniques, in both its low- and medium-voltage applications [17, 18].

Deep oscillation therapy is an effective technique for pain relief and has the advantage of being deep-acting and achieved with minimal mechanical impact, meaning it is useful in acute pain processes [24, 25].

Medium-voltage electrotherapy, like interferential current therapy, combined with ultrasound with zeroed out parameters, together known as pain tracking, is applied in order to diagnose or locate the trigger points. It has therapeutic and prognostic value, enabling detection of significant changes from one session to the next, and has demonstrated positive results in the immediate relief of acute radicular and neurogenic pain, as well as somatic pain [17, 18]. Therefore, in this work we intend to evaluate the usefulness of the pain tracking technique in acute mechanical low back pain, as well as the evolution of pain using a visual analogue scale, disability based on the Waddell test before and after treatment, and the onset of clinical changes in this group of patients.

## 2. Method

We performed an experimental prospective (longitudinal) explanatory study between January 2011 and September 2012.

The universe was made up of all those patients referred to the Physical Medicine and Rehabilitation Clinic at the Clinical Research Center with clinically diagnosed acute mechanical low back pain. The sample was made up of 90 patients who expressed a desire to participate in the study and whose low back pain had begun no more than 7 days ago, presenting neither radiation to the lower body parts nor decompensation. Defectual psychiatric and psychotic patients were excluded from the study as were those whose state of health prevented them from participating or where medical treatment was indicated. All patients were interviewed and physically examined by palpation of the paravertebral region, checking for contracture and pain in the spinous processes of the lumbar vertebrae in order to diagnose minor intervertebral derangement (MID). A medical history was taken, including sociodemographic and epidemiological characteristics, and the visual analogue scale (VAS) and Foral Waddell test were applied before and after treatment with physical agents.

The sample was divided into 2 groups, where Group I, or the study group, corresponded to even numbers and Group II, or the control group, to odd numbers. By way of treatment plan, patients received daily treatment over 7 sessions.

The study group was treated using the pain tracking technique with interferential current applied to the lumbosacral spine region, with amplitude modulation frequency (AMF) of 100 Hz, spectrum of 0 Hz, carrier frequency of 4000 Hz, and ultrasound with zeroed out parameters. The positive electrode was used as the indifferent electrode and positioned on the metamera itself or nearby target area, while the negative electrode was substituted for the ultrasound applicator and moved slowly over the target area. Treatment began at the lowest intensity, increasing progressively until reaching the limit of tolerance. The patient was put in ventral decubitus (prone) position with a pillow under the abdomen.

Group II, or the control group, received treatment with interferential current and cryotherapy. The patient was put in the lateral or ventral decubitus (prone) position with a pillow under the abdomen to correct lumbar lordosis. Interferential current was applied using the tetrapolar method to the lumbosacral spine region, with carrier frequency of 4000 Hz, AMF of 100 Hz, spectrum of 50 Hz, with spectrum variance 6/6, for 10 minutes.

We used PHYSIOMED's IONOSON-Expert device for both groups.

After the electrotherapy, in both groups we applied 10 minutes of cryotherapy with a clockwise massage over the 7 treatment sessions. Both groups were issued with postural hygiene recommendations at home.

In terms of the professions of our patients, 27 are labourers, 17 are professionals, 11 are housewives, and 10 are drivers. The remainder are office workers or students.

In the study, pain was triggered by prolonged static posture in the workplace for 31 patients, intense physical labour for 26 patients, bending and twisting and forward trunk bending for 15 and 12, respectively, and 6 through carrying out work with vibrations. We found that patients worsened sitting in 23 cases, with trunk movements in 22 and standing in 18. In terms of postures that provided relief, the lateral decubitus position was reported by 54 patients as being effective, the Williams position by 29 patients, and the ventral decubitus (prone) position by 7 patients.

Some 83.3% of the patients had contracture and minor intervertebral derangement (MID) was detected in the physical examination of 26 patients.

*Evaluation Criteria.* Pain was evaluated according to its intensity using the* visual analogue scale* by assigning it a score:

- No pain = 0.
- Slight pain = 1–3.
- Moderate pain = 4–6.
- Intense pain = 7–10.

For the evaluation of the Foral Waddell test, we used the following scale:

- No disability = 0.
- Slight disability = 1–3.
- Moderate disability = 4–6.
- Severe disability = 7–9.

A medical history was taken for each patient. A database was created to process these medical histories using descriptive techniques (median). The results are expressed in tables.

To establish a comparison between the pain variables, the Wilcoxon signed-rank test and the disability test were applied to both groups before and after treatment, and the Mann Whitney test was used to compare the onset of analgesia also in both groups.

The tests were performed with a predetermined significance level of *p* ≤ 0.05.

## 3. Results

Table 1 shows the evolution of pain based on VAS before and after treatment in both groups. The median score at start of treatment in both groups was 7.00, with no significant differences between the groups for *p* = 0.597. At the end of treatment, the median VAS score was 0.00 in Group I and 1.00 in Group II with *p* < 0.037, with significant differences between the two groups, meaning that both treatments were effective in the treatment of acute mechanical low back pain.

When we compared the pain based on its clinical evolution from start to end of treatment, we observed highly significant differences in the two groups with *p* < 0.001.

Table 2 presents patient evolution based on the Waddell test. In the study group, the median was 5.00 at the start of treatment and 0.00 by the end of treatment with *p* = 0.823, while, in the control group, the median was 5.00 at the start of treatment and 0.00 by the end of treatment with *p* = 0.131, with no significant differences between the groups. When we compared the test at the start and end of treatment by groups, we observed highly significant differences in the two groups with *p* < 0.001.

We applied the Mann Whitney test to compare the onset of analgesia between the groups. The median score in the study group was 2.00, while in the control group it was 4.00. Scores ranged between 1 and 6 in both groups, observing highly significant differences with *p* < 0.001, where pain relief was faster among the patients who were treated using the pain tracking technique (see Table 3).

## 4. Discussion

Currently peripheral electrical analgesia is considered the least risky therapy for pain treatment. It has been demonstrated that interferential current relieves pain by stimulation of the large diameter myelinated fibers, according to Melzack and Wall's “gate control” theory. To stimulate this mechanism, a frequency higher than 100 Hz must be used at a low intensity below the pain threshold [17, 18]. In the case of pain tracking, the effect of the ultrasound is added to the locating of pain points and production of an intense sensitive stimulus with a fixed modulated frequency of 100 Hz, providing immediate pain relief. This is why the author believes that, given the demonstrated analgesic effect of medium-voltage electrotherapy, pain relief was achieved in both groups and in the pain tracking group the effect of the ultrasound meant the improvements were faster than when just interferential current was applied.

It is important to remark that we used interferential current for this study because of its advantages over low-voltage currents: stimulation is not aggressive, galvanic effects are eliminated, it is more deep-acting, and there is no unpleasant sensation when the circuit is opened [17, 18].

In reviews of EBSCO, LILACS, MEDLINE, Cochrane Library, and PubMed, we found no published study on the usefulness of the pain tracking technique in acute mechanical low back pain. The mesh (medical subject headings) search terms were “pain tracking.”

Hoek (1972) indicates that the incidence of occupational disability through back pain increased from 2.9% in 1959 to 61% in 1968 in the Dutch population. Nachelsom (1992) estimates a rise within the Swedish population from 3% in 1980 to 8% in 1990 and of 25 days absent from work in 1983 to 30 days in 1985 and 40 days in 1990. Waddell (1987) reports an increase within the Canadian population from 2.17% in 1953 to 5.46% in 1998. Uy tlendael (1981) reports an increase within the Belgian population from 0.4% in 1971 to 3.6% in 1978. It is observed that while the rate of low back pain has risen arithmetically, the rate of disability resulting from back pain has risen geometrically [2, 26].

Low back pain renders the patient unable to carry out certain activities. However, this disability is significantly modified when the pain is relieved, and the patient is able to resume those activities they had had to stop with the onset of the pain.

## 5. Conclusions

Both the pain tracking and interferential current techniques combined with cryotherapy are useful treatments for acute mechanical low back pain.

The onset of analgesia is faster when using the pain tracking technique.

## Figures and Tables

**Table 1:** Evolution of pain based on VAS in both groups at the start and end of treatment.

VAS	Group I Median	Group II Median	
Start	7.00	7.00	*p* = 0.597
End	0.00	1.00	*p* < 0.037
	*p* < 0.001	*p* < 0.001	

Source: SPSS database.

**Table 2:** Waddell test on both groups at start and end of treatment.

Waddell test	Group I Median	Group II Median	
Start	5.00	5.00	*p* = 0.823
End	0.00	1.00	*p* = 0.131
	*p* < 0.001	*p* < 0.001	

Source: SPSS database.

**Table 3:** Onset of analgesia in both groups.

	Onset of analgesia Median	Minimum	Maximum
Group I	2.00	1	6
Group II	4.00	1	6

Source: SPSS database, *p* < 0.001.
